# Complete Genome Sequence of a Novel *Sulfolobales* Archaeon Strain, HS-7, Isolated from the Unzen Hot Spring in Japan

**DOI:** 10.1128/MRA.00582-21

**Published:** 2021-09-23

**Authors:** Hiromi Omokawa, Norio Kurosawa, Shingo Kato, Takashi Itoh, Moriya Ohkuma, Hiroyuki D. Sakai

**Affiliations:** a Department of Environmental Engineering for Symbiosis, Graduate School of Science and Engineering, Soka University, Hachioji, Tokyo, Japan; b Department of Science and Engineering for Sustainable Innovation, Faculty of Science and Engineering, Soka University, Hachioji, Tokyo, Japan; c Japan Collection of Microorganisms, RIKEN BioResource Center, Tsukuba, Ibaraki, Japan; Indiana University, Bloomington

## Abstract

The order *Sulfolobales* includes thermoacidophilic archaea that thrive in acidic geothermal environments. A novel *Sulfolobales* archaeon strain, HS-7, which may represent a novel genus, was isolated from an acidic hot spring in Japan. We report the 2.15-Mb complete genome sequence of strain HS-7.

## ANNOUNCEMENT

Members of the order *Sulfolobales* are often isolated from acidic geothermal environments. Several novel species have recently been proposed in the order *Sulfolobales* with reclassification of previous taxa (1–3), expanding our knowledge of the phylogenetic and genomic diversity in this taxon. We isolated another novel strain, HS-7. Here, we report its complete genome sequence.

Muddy water was collected in a 100-ml glass bottle at the Unzen hot spring in Japan (32°44′23″N, 130°15′54″E) (65°C to 68.5°C, pH 2.3). An aliquot of the sample was inoculated into modified Brock′s basal salt (MBS) medium (4) with 0.5 g/liter glucose (pH 1.5, 65°C) to perform enrichment culture. Strain HS-7 was isolated from the enrichment culture by dilution to extinction following the protocol described previously (5) except for the medium. For DNA extraction, HS-7 was cultivated in MBS medium with 1 g/liter tryptone. One liter of the culture was centrifuged (15,000 × *g* at 25°C for 15 min), and DNA extraction was performed using the Genomic-tip 100/G kit (Qiagen). This DNA was used for all DNA library preparations. The DNA library was prepared with a QIAseq FX DNA library kit (Qiagen) or a NEBNext Ultra II FS DNA library preparation kit for Illumina (New England BioLabs). Short-read sequencing was conducted on MiSeq (301-bp paired-end reads) and NovaSeq 6000 (150-bp paired-end reads) platforms, resulting in a total of 13,589,534 raw short reads (3,121,051,187 bp). The reads were quality filtered using fastp v.0.20.1 (6), resulting in a total of 7,151,146 quality-filtered reads (945,534,048 bp). Long-read sequencing was performed using a MinION platform with an R9 flow cell, SQK-LSK109, and EXP-NBD104 (Oxford Nanopore Technologies [ONT]) following the protocol described by ONT (NBE_9065_v109_revZ_14Aug2019). DNA fragments of 3 kb or longer were enriched by the protocol. After base calling by MinKNOW v.4.2.8 (7), a total of 428,793 raw long reads (1,521,767,622 bp [*N*_50_, 9,792 bp]) were obtained. The reads were quality filtered using Filtlong v.0.2.0 (https://github.com/rrwick/Filtlong), resulting in a total of 213,403 quality-filtered reads (1,000,002,879 bp [*N*_50_, 6,584 bp]). Using all of the quality-filtered reads, genome assembly was performed by Unicycler v.0.4.8 (8), followed by annotation with DFAST v.1.4.0 (9). Default parameters were used for all software.

**FIG 1:**
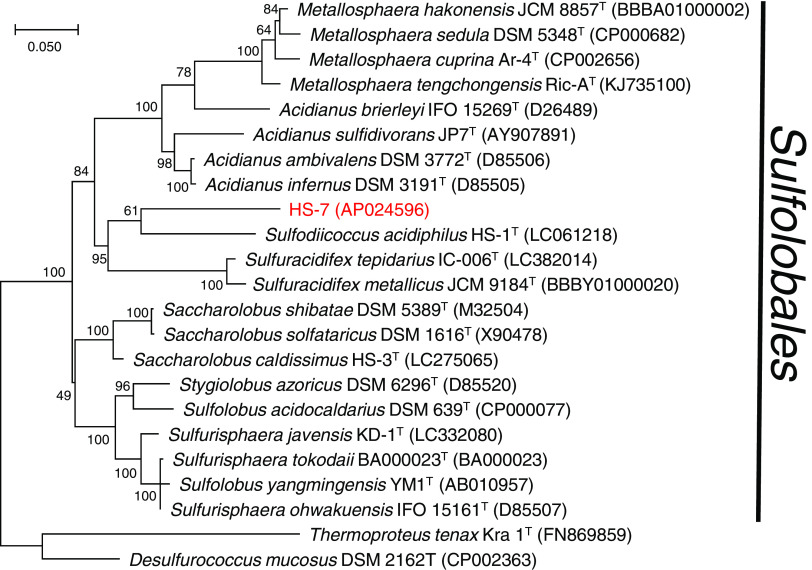
Maximum-likelihood phylogenetic tree of 16S rRNA gene sequences. The sequence alignment was conducted by the MUSCLE program implemented in MEGA X (10), with default parameters. GenBank accession numbers are indicated in parentheses.

A circular contig of 2,151,177 bp was obtained (GC content, 39.6%). The genome contained 2,140 coding sequences, a single copy of the rRNA operon, and 46 tRNAs. The maximum-likelihood tree of the 16S rRNA gene constructed by MEGA X (10) showed that strain HS-7 belongs to the order *Sulfolobales* (Fig. 1). However, the 16S rRNA sequence similarity calculated by BLASTN was less than 88% in comparison with any type strain in the order *Sulfolobales*, suggesting that HS-7 should be assigned to a novel genus in this taxon.

In this report, we determined the complete genome sequence of an archaeon probably representing a novel genus in the order *Sulfolobales.* The data contribute to our understanding of the genomic organization and diversity in the order *Sulfolobales.*

### Data availability.

The genome sequence of strain HS-7 and raw reads have been deposited in DDBJ/ENA/GenBank under the accession numbers AP024596, DRR286851, DRR286852, and DRR286853.
